# Pulmonary cystic destruction in systemic sclerosis and antisynthetase syndrome associated non-specific interstitial pneumonitis: a case series of a novel radiological phenotype

**DOI:** 10.1093/rap/rkag030

**Published:** 2026-03-24

**Authors:** Matthew Wells, Giles Dixon, Nidhi Bhatt, Jo Hardy, Huzaifa I Adamali, Harsha Gunawardena, John D Pauling, Shaney L Barratt, Anthony Edey

**Affiliations:** Bristol Interstitial Lung Disease Service, North Bristol NHS Trust, Bristol, UK; Department of Clinical Specialties and Related Sciences, University of Bristol, Bristol, UK; Bristol Interstitial Lung Disease Service, North Bristol NHS Trust, Bristol, UK; Bristol Interstitial Lung Disease Service, North Bristol NHS Trust, Bristol, UK; Department of Pathology, North Bristol NHS Trust, Bristol, UK; Bristol Interstitial Lung Disease Service, North Bristol NHS Trust, Bristol, UK; Bristol Interstitial Lung Disease Service, North Bristol NHS Trust, Bristol, UK; Bristol Interstitial Lung Disease Service, North Bristol NHS Trust, Bristol, UK; Bristol Interstitial Lung Disease Service, North Bristol NHS Trust, Bristol, UK; Translational Health Sciences, Bristol Medical School, University of Bristol, Bristol, UK; Bristol Interstitial Lung Disease Service, North Bristol NHS Trust, Bristol, UK; Translational Health Sciences, Bristol Medical School, University of Bristol, Bristol, UK; Bristol Interstitial Lung Disease Service, North Bristol NHS Trust, Bristol, UK; Department of Radiology, North Bristol NHS Trust, Bristol, UK

**Keywords:** systemic sclerosis, anti-synthetase syndrome, interstitial lung disease, cystic destruction, NSIP

## Abstract

**Objectives:**

To describe a novel radiographic phenotype of systemic autoimmune rheumatic disease–associated interstitial lung disease (SARD-ILD) observed within a regional specialist multidisciplinary (MDT) service.

**Methods:**

This was a retrospective case series of patients with SARD-ILD complicated by radiographic pulmonary cystic destruction. Cases were identified through review of MDT records from the North Bristol SARD-ILD service. Cases with MDT reported ‘cystic changes’ were identified and clinico-radio-pathological features reviewed.

**Results:**

Five cases of SSc-associated ILD (SSc-ILD) and two cases of antisynthetase syndrome–associated ILD (ASyS-ILD) with cystic changes were identified among a total of 108 SARD-ILD patients. All seven cases presented with radiological patterns of cellular non-specific interstitial pneumonia (NSIP). The median age at diagnosis with SARD was 33 years and six cases had ILD identified within 6 months of SARD diagnosis. The cohort was ethnically diverse, with one ex-smoker. Microcystic destructive changes appeared and progressed within areas of ground glass despite standard-of-care immunomodulation. Changes are radiographically and histologically distinct from traction bronchiolectasis, honeycombing and smoking-related lung disease. Histological specimens were available for two cases confirming fibrotic NSIP, with one including affected tissue demonstrating intimal thickening of the pulmonary vasculature. Over a median follow-up of 49 months, all cases remain alive and transplant free; five fulfilled criteria for progressive disease and six required ambulatory oxygen.

**Conclusions:**

This represents the first western cohort describing microcystic destructive pulmonary changes in SSc-ILD and the first report in ASyS-ILD. Systematic case identification is required to determine demographic associations and prognostic implications.

Key messages

- A novel radiographic phenotype of SARD-ILD is presented, consisting of progressive microcystic destruction complicating non-specific interstitial pneumonitis (NSIP). Aetiology and impact upon prognosis remain uncertain; changes appear independent of cigarette smoke exposure.
- Future work including a control population is necessary to determine any demographic or prognostic associations.

## Introduction

Systemic sclerosis (SSc) and antisynthetase syndrome (ASyS) are systemic autoimmune rheumatic diseases (SARDs) commonly associated with interstitial lung disease (ILD). SSc is uncommon, with a prevalence of 235.5/million in the UK [1]; ASyS is rarer still, with uncertain prevalence data due to a historic lack of recognised classification criteria and evolving definitions of disease [2, 3].

ILD occurs in 35–53% of SSc cases, representing the leading cause of mortality, and affects more than two-thirds of ASyS patients [4, 5]. Radiological SARD-ILD patterns include predominantly non-specific interstitial pneumonitis (NSIP) in SSc, with usual interstitial pneumonia (UIP) and organising pneumonia (OP) also reported [6, 7].

Radiological descriptions for coexistent fibrosis and emphysema vary widely, with terms such as ‘combined pulmonary fibrosis and emphysema’ (CPFE), ‘smoke-related interstitial fibrosis’ (SRIF) and ‘airspace enlargement with fibrosis’ (AEF) in use [8–10]. Some appear to relate to SARD, but this is complex. Descriptive inconsistencies are exemplified by a 2021 systematic literature review that included six studies assessing outcomes in SARD-associated CPFE, with vastly different radiological inclusion criteria [11].

Following from preliminary work in which ‘cystic’ changes were identified at a frequency of 35% among a cohort of 40 SSc-ILD patients, a 2018 Japanese study described the clinico-radio-pathological features of SSc-ILD with a focus on ‘pulmonary emphysema’ [12, 13]. Authors identified 16/21 SSc cases with an unusual pattern of ‘pathological-pulmonary emphysema’ predominantly observed within cases with high-resolution CT (HRCT) findings consistent with NSIP-pattern ILD [13]. Five cases were characterised by thin-walled areas of low attenuation appearing adjacent to interstitial abnormalities, distinct from other descriptions of ‘emphysema’, ‘CPFE’ and end-stage fibrosis reported within SSc populations [12, 14]. These findings, coupled with the relatively low prevalence of ever-smokers among those with pathological pulmonary emphysema (62.5%), raised suspicion for alternative aetiologies, with authors proposing a potential microvascular contribution to the appearances on the basis of histological vascular abnormalities and concluding that their findings represent a novel radio-pathological feature of SSc-ILD [13].

We present the first Western case series of seven SARD patients displaying similar pulmonary cystic destruction on CT, five of whom have SSc and two ASyS, a separate condition in which these changes have not previously been described. These changes appear to represent novel features that may be indicative of a broader spectrum of radio-pathological features predominating in SSc, but potentially also seen across SARD-ILD, including ASyS.

## Methods

A retrospective case series of patients with SARD-ILD complicated by concomitant or evolving pulmonary cystic destructive changes is presented. Cases were identified through a comprehensive review of the North Bristol regional SARD-ILD multidisciplinary team (MDT) meeting records, censored to 1 June 2025. All cases with an MDT diagnosis of SSc, ASyS, mixed or overlap connective tissue disease (M-CTD) or Sjogren disease (SD) were identified and sequential MDT records reviewed for all identified cases. Cases with outcomes reporting on the presence of ‘cystic’ changes were identified and medical records, imaging and clinical reporting systems were reviewed for clinico-radio-pathological features.

A descriptive analysis was performed with data censored to 1 June 2025. Progression was defined in line with the INBUILD trial (NCT02999178) [14].

All images were reviewed on 1–1.25 mm volumetric datasets with a high-resolution spatial kernel on lung windows by an experienced thoracic radiologist and ILD physician (A.E. and S.L.B.) with reference to the American Thoracic Society imaging guidelines [15, 16]. Ethical approval was not required for this case series, in accordance with UK Health Research Authority guidance, as this study represents a retrospective description of routine clinical practice. All patients provided written informed consent for inclusion and publication.

## Results

The records review identified seven patients with ‘cystic’ changes reported on HRCT or MDT records, following assessment of 108 separate SARD-ILD cases (46 SSc-ILD, 39 ASyS-ILD, 12 M-CTD, 11 SD), a prevalence of 6.5% among this regional SARD-ILD cohort. Of the seven identified cases, five had a diagnosis of SSc fulfilling modern classification criteria and two had a diagnosis of ASyS, with one fulfilling proposed classification criteria for ‘definite’ ASyS [17, 18].

**Figure 1:**
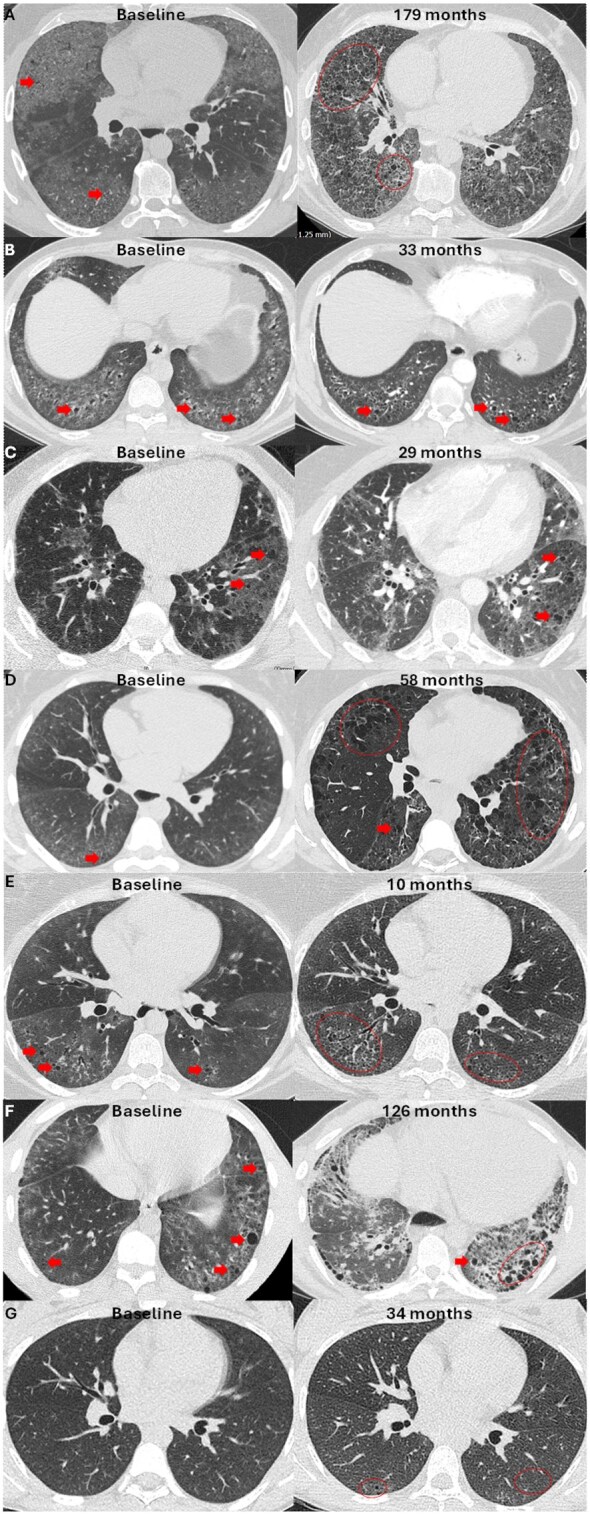
CT images at baseline/diagnosis with SARD (left) and follow-up (right). **(A–F)** Baseline images show ground glass opacification typical of cellular NSIP pattern lung disease with early internal cystic destruction evident within areas of ground glass. **(E)** Minor reticular changes within the ground glass and incidental pneumomediastinum. **(G)** The absence of ILD at presentation. Follow-up images show varying degrees of progression with more conspicuous destructive cystic changes most evident in A–C. Cystic destruction is more subtle in E and G. Background established fibrosis is evident in A and F at follow-up yet is discrete from cystic destruction. All baseline images were performed <6 months after diagnosis with SARD. Unannotated images are available (Supplementary Fig. S3).

Cases had a median follow-up of 49 months (range 10–191). Five had a radiological cellular NSIP pattern of ILD identified within 3 months of presentation with either SSc or ASyS, with varying extents of cystic destruction present on baseline HRCT (Fig. 1A–F). One case of SSc had cellular NSIP with cystic destruction detected on baseline HRCT scan 6 months after SSc diagnosis; a separate SSc case had no ILD evident on baseline HRCT at presentation with SSc (Fig. 1G), with cellular NSIP diagnosed 20 months later. Early cystic destructive changes became apparent by 34 months from SSc diagnosis (14 months from ILD diagnosis) (Fig. 1H).

Clinico-radio-pathological characteristics are summarised in Table 1. The cohort was ethnically diverse, comprising three Black patients, two Caucasian patients, one South Asian patient and one Hispanic patient. The median age at SARD and ILD diagnosis was 33 years. There was only one ever-smoker (ex-smoker with a 25 pack-year history).

**Table 1:** Demographics and clinic-radio-pathological details of included cases.

Characteristics	SSc (*n* = 5)	ASyS (*n* = 2)	Total (*N* = 7)
Age at SARD diagnosis, years, median (range)	25 (15–35)	40 (37–43)	33 (15–43)
Age at ILD diagnosis, years, median (range)	25 (15–35)	40 (37–43)	34 (15–43)
SSc subtype, *n*			
Diffuse	2/5	–	2/5
Limited	3/5	–	3/5
Sex, *n*			
Male	2/5	2/2	4/7
Female	3/5	0/2	3/7
Ethnicity, *n*			
Black	2/5	1/2	3/7
Caucasian	2/5	0/2	2/7
Hispanic	1/5	0/2	1/7
South Asian	0/5	1/2	1/7
Smoking status, *n*			
Ex-smoker	0/5	1/2	1/7
Never smoker	5/5	1/2	0/7
Timing of ILD diagnosis, *n*			
ILD predates SARD diagnosis	0/5	0/2	0/7
ILD identified ≤3 months from SARD diagnosis	3/5	2/2	5/7
Time from SARD diagnosis to ILD diagnosis, months, median (range)	1 (0–20)	0	0 (0–20)
Radiology			
Radiological ILD pattern, *n*			
NSIP (cellular)	5/5	2/2	7/7
Other	0/5	0/2	0/7
Time to cystic destruction appearance, *n*			
Evident on baseline HRCT	4/5	2/2	6/7
During follow-up (after SARD diagnosis)	1/5	0/2	1/7 (34)
Physiology			
Baseline physiology (% predicted)			
FVC, median (range)	72 (68–84)	73 (62–84)	78 (62–84)
DLCO, median (range)	37 (26–57)	42 (34–50)	37 (26–57)
6-min walk test desaturation <90%, *n*	0/1	0/2	0/3
Latest physiology			
FVC, median (range)	60 (36–83)	73 (67–79)	65 (36–83)
DLCO, median (range)	38 (15–45)	44 (43–44)	40 (15–45)
6-min walk test desaturation <90%, *n*	4/5	2/2	6/7
Cytology/histology			
Bronchoscopy, *n*	2/5	1/2	3/7
Cytology			
Alveolar macrophages, %, median (range)	70 (60–80)	40	60 (40–80)
Lymphocytes, %, median (range)	7.5 (5–10)	10	10 (5–10)
Neutrophils, %, median (range)	10 (10–10)	20	10 (10–20)
Eosinophils, %, median (range)	12.5 (0–25)	30	25 (0–30)
Microscopy and culture			
No organisms, *n*	2/2	1/1	3/3
VATS biopsy, *n*	2/5	0/5	2/7
Fibrotic NSIP histology, *n*	2/2	–	2/2
Serology			
Autoantibody status, *n*			
Anti-SCl70	5/5	0/2	5/7
Anti-PL7	0/5	1/2	1/7
Seronegative	0/5	1/2	1/7^a^
Anti-phospholipid status, *n*			
Anti-cardiolipin	0/4	0/1	0/5
Lupus anticoagulant	0/3	0/1	0/4
Beta-2-glycoprotein 1	0/1	0/1	0/2
ANCA status, *n*			
Negative	1/3	0/2	1/5
pANCA	2/3^b^	0/2	2/5
cANCA	0/3	1/2	1/5
Atypical ANCA	0/3	1/2	1/5
Treatment			
Prior/current immunomodulatory treatment, *n*			
MMF	5/5	2/2	7/7
CYC	2/5	0/2	2/7
RTX	3/5	1/2	4/7
TOC	1/5	0/2	1/7
Antifibrotic treatment, *n*			
Nintedanib	4/5	0/2	4/7
Outcomes			
Clinical follow-up, months, median (range)	64 (10–191)	42 (35–49)	49 (10–191)
Radiological follow-up, months, median (range)	42 (10–179)	31 (29–33)	33 (10–179)
Transplant-free survival, *n*	5/5	2/2	7/7
Progression-free survivalc, *n*	2/5	0/2	2/7
Time until progression, months, median (range)	45 (36–55)	27 (25–28)	36 (25–55)
Qualifies for supplementary O_2_ (uses), *n*	4/5 (1/4)	2/2 (0/2)	6/7 (1/6)
Time until eligible for supplementary O_2_, months, median (range)	31 (6–50)	16 (13–19)	22 (6–50)
Hospitalisation (respiratory cause), *n*	1/5	1/2	2/7

cANCA: cytoplasmic ANCA; CYC: cyclophosphamide; DLCO: diffusion capacity of the lung for carbon monoxide; FVC: forced vital capacity; MMF: mycophenolate mofetil; pANCA: perinuclear ANCA; RTX: rituximab; TOC: tocilizumab.

a Seronegative patient had a cytoplasmic staining pattern evident on immunofluorescence and identifiable antibodies on immunoprecipitation.

b One patient developed ANCA-MPO-positive renal vasculitis 13 years after initial presentation with SSc-ILD. Vasculitis was ascribed to nintedanib use. There was no prior ANCA test result available.

c Progression-free survival defined as any of FVC decline of ≥10%, FVC decline of 5–<10% with worsening symptoms, FVC decline of 5–<10% with worsening fibrosis on HRCT or worsening of fibrosis on HRCT with worsening symptoms [14].

Lung function demonstrated restrictive impairment with reduced lung capacity for diffusion of carbon monoxide (TLCO, Table 1) at presentation. Development of exertional desaturation on hall walk testing was common, occurring amongst six patients during follow-up, a median of 22 months after ILD diagnosis.

Of the five cases with SSc, two had a diffuse cutaneous phenotype and three had a limited cutaneous phenotype. A modified Rodnan skin score within 1 month of diagnosis was available for two SSc patients (4 and 40/51). Vascular features included RP in six cases; three of which have been complicated by digital ulceration during follow-up.

All SSc cases were anti-topoisomerase positive and ASyS cases were anti-PL7 positive or positive for unknown antibodies on immunoprecipitation assay with a corresponding cytoplasmic immunofluorescence pattern. Five had aPL testing available (all negative). ANCA test results are detailed in Table 1: one case tested positive for perinuclear ANCA with myeloperoxidase activity in the context of renal vasculitis 13 years following diagnosis with SSc-ILD, without previous ANCA testing.

The basal dominant cystic changes appeared within areas of interstitial ground glass opacity, consistent with cellular NSIP distribution (Fig. 1). Cysts ranged from 2 to 10 mm and were distributed throughout the involved parenchyma. Destruction progressed and increased over time. Honeycombing and traction bronchiolectasis were excluded based on distribution (central rather than subpleural) and volumetric assessment, respectively.

Two cases had right heart catheterisation (RHC) performed; one was diagnosed with class III pulmonary hypertension and the other was normal. No other case has had biochemical or echocardiographic features suggestive of pulmonary hypertension (PH).

Three cases (two SSc, one ASyS) had bronchoscopy with bronchoalveolar lavage (BAL) performed, with alveolar macrophages the predominant cell type in all, but with neutrophils, lymphocytes and eosinophils also identified (Table 1). Bronchoscopy was performed to determine inflammatory burden to guide further immunosuppression in each case rather than to investigate for infection. Microscopy and culture of BAL fluid was negative.

Two of these three (both SSc) also had subsequent videoscopic-assisted thoracic surgical (VATS) biopsies performed 12 and 57 months after ILD diagnosis. Findings were consistent with fibrotic NSIP lung disease without features of UIP or emphysema. Only the latter sample included an area of cystic destruction and was notable for severe pulmonary arterial luminal narrowing secondary to intimal thickening (Supplementary Fig. S1).

All patients have been established on mycophenolate mofetil, with several receiving additional cyclophosphamide or biologic therapy (Table 1). Four cases have received nintedanib for progressive pulmonary fibrosis, with one stopping treatment due to the development of renal vasculitis. Progression of cystic changes occurred despite therapy.

All cases remain alive and transplant free over the available duration of follow-up. Two cases were referred for transplant assessment but were declined given gastro-oesophageal reflux disease. While six patients are eligible for ambulatory oxygen prescription, only one patient is currently prescribed this (patient choice). Two patients have required hospitalisation for a cardiorespiratory indication since diagnosis (lower respiratory tract infections). Five patients have fulfilled criteria for progression a median of 36 months following diagnosis (range 25–55).

## Discussion

To our knowledge, only a single case report and a separate case series describe similar changes in SSc-ILD, both originating from Japan [13, 19]. This may reflect the lack of consistent descriptive terminology, with such changes possibly having been reported elsewhere using different radiological descriptors.

Our cohort is notable for its ethnic diversity and absence of obstructive spirometry, bullous or paraseptal destruction and smoking histories, suggesting causes other than cigarette smoke exposure.

Yamakawa *et al.* [13] proposed a vascular aetiology for the observed ‘pathological-pulmonary emphysema’ based on findings of abnormal vasculature identified on widely available biopsy specimens within their cohort, although these changes were not omnipresent. We observed similar changes in one of the two VATS biopsy specimens available (Supplementary Fig. S1). While the second specimen did not show vascular abnormalities, this did not include cystic parenchyma (based on postoperative review of the CT to locate the biopsy site). Although pulmonary vascular changes are well described in SSc-ILD [20], this is usually within the context of demonstrable class I PH and no included cases have evolved this. While only two have had RHC performed, all receive regular surveillance during follow-up, consisting of N-terminal prohormone of brain natriuretic peptide (NT-proBNP) and surveillance echocardiograms with referral for RHC if indicated.

Another consideration is that the observed cystic destruction reflects long-standing pulmonary inflammation with subsequent tissue damage. Where available, BAL fluid has demonstrated the presence of inflammatory cells. However, in the absence of larger cohorts and control populations with widely available cytology and histology, no concrete conclusions can be drawn as to the aetiopathogenesis of these changes.

The observed progressive microcystic destructive changes differ from typical smoking-related changes in SARD-ILD, which tend to manifest as upper zone paraseptal or centrilobular emphysema with basal fibrosis (Supplementary Fig. S2). They are also distinct from UIP pattern fibrosis and traction airway dilatation, with cystic areas developing early in the disease course within central areas of cellular NSIP inflammation rather than within the basal and subpleural distribution typical of UIP.

This retrospective observational case series has multiple limitations that need to be acknowledged. First, case identification relied upon MDT capture of ‘cystic’ changes introducing a potential selection bias. By reviewing SARD-ILD MDT records alone without a systematic review of HRCT scans, we rely on reporting radiologist capture of such changes as well as overlook the possibility of identifying such changes within other forms of NSIP, including idiopathic. If present across the wider spectrum of NSIP, this may suggest that changes reflect an end-stage of cellular NSIP. The extremely small number of identified cases, presence of missing data and lack of a comparator cohort limits generalisability and the ability to draw conclusions with respect to prevalence, affected patient demographics and prognosis.

Comments on the aetiology of these changes remain entirely speculative, limited by the lack of universal invasive diagnostic procedures, including bronchoscopy, lung biopsy and RHC. Three cases were regional referrals to our tertiary centre, and local imaging protocols may include subtle differences. The inclusion of two cases of ASyS, described for the first time in the literature, is essentially anecdotal.

Our findings highlight a progressive microcystic ILD pattern seen in SARD-associated NSIP that progresses despite immunosuppressive and antifibrotic therapies, described for the first time in a UK SSc cohort as well as for the first time ever in ASyS, a distinct SARD with differing pathophysiology. Despite separate underlying SARD diagnoses, cases are unified by a radiological pattern of cellular NSIP.

There is an unmet need for harmonious radiological reporting and terminology, the lack of which may limit the generalisability of our findings. Early recognition of this form of SARD-related ILD is important. Future work to identify larger cohorts and include comparator SSc and SARD-ILD populations is desirable to examine any demographic associations and impact upon prognosis and morbidity, as well as more systematic case finding strategies to explore whether these changes are unique to SARD or can be seen across the cellular NSIP spectrum. Prospective studies of SARD-ILD and cellular NSIP may be helpful in establishing the natural history and evolution of such changes.

## Supplementary Material

rkag030_Supplementary_Data

## Data Availability

Data cannot be shared due to patient confidentiality agreements. The data are strictly confidential to protect the privacy of the participants.
