# Supplementary material for: Pulmonary cystic destruction in systemic sclerosis and antisynthetase syndrome associated non-specific interstitial pneumonitis: a case series of a novel radiological phenotype
Source: Rheumatol Adv Pract. 2026 Mar 24;10(2):rkag030. doi: 10.1093/rap/rkag030 (PMC13070459; doi:10.1093/rap/rkag030)
Supplement: rkag030_Supplementary_Data [file rkag030_supplementary_data.docx]

 
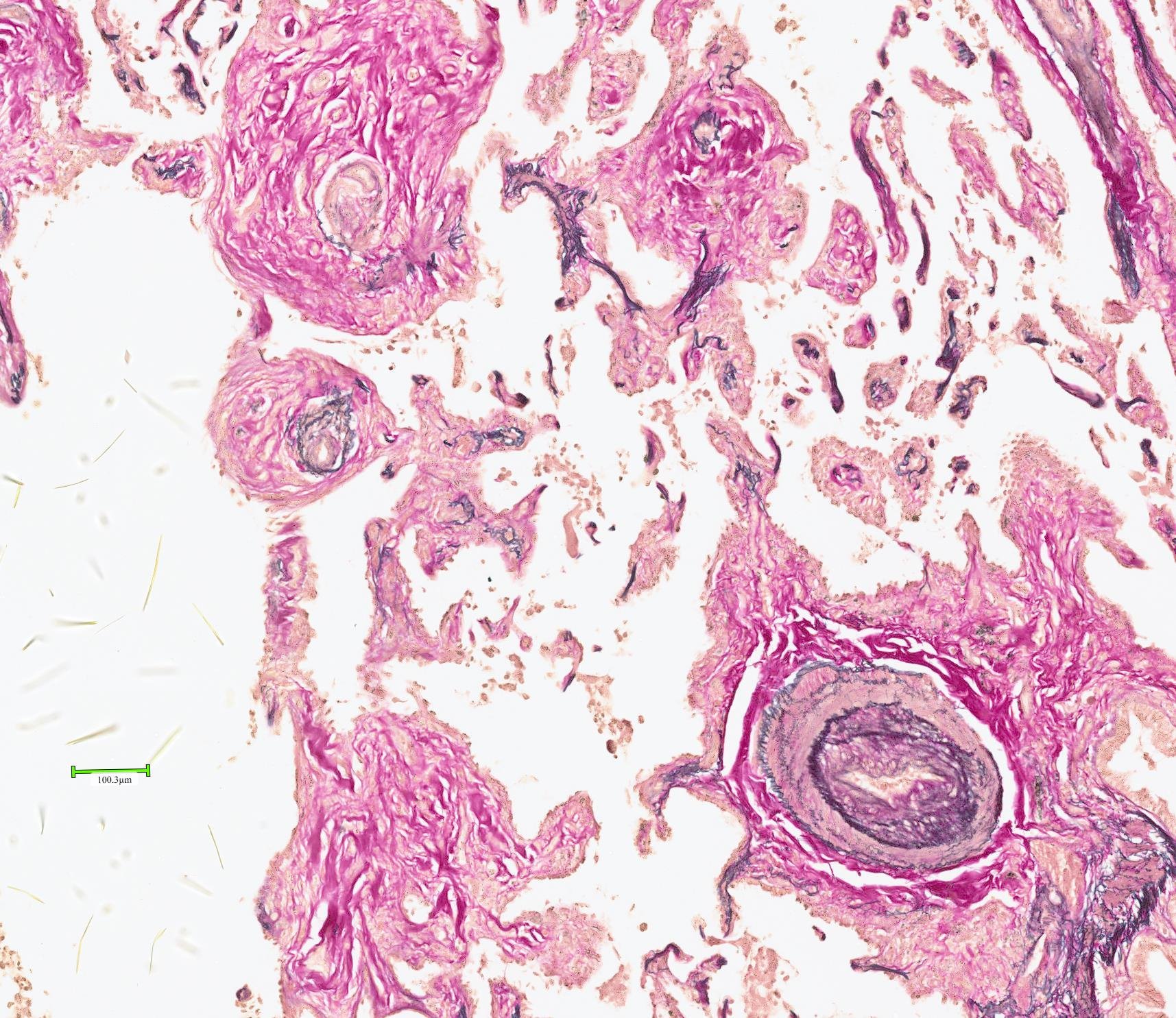


Supplementary Figure S1. Lung tissue from right middle/lower lobe obtained via VATS biopsy 59 months following diagnosis of SSc -ILD in a patient with cystic destructive changes (case A in figure 1). Elastin van Gieson staining highlights thickened arterial walls (arrows). Biopsy did not show features of UIP or emphysema.

Alt text: Histology slide showing intimal thickening of pulmonary arteries (black arrows).


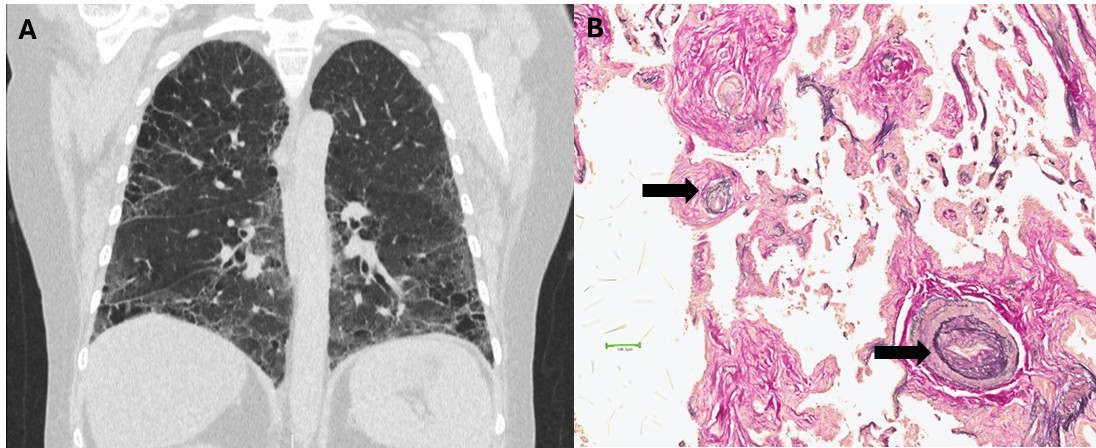


Supplementary Figure S2. HRCT coronal images from a patient with classifiable anti-SCl70 positive SSc and a smoking history of 20 pack years. Upper zone predominant paraseptal emphysema typical of cigarette smoke exposure with basal macrocystic honeycombing in keeping with a definite UIP pattern of fibrosis. Initial presentation was with cellular NSIP 120 months earlier.

Alt text: Coronal HRCT images of a patient with changes typical of smoking related emphysema and also basal UIP pattern fibrosis.


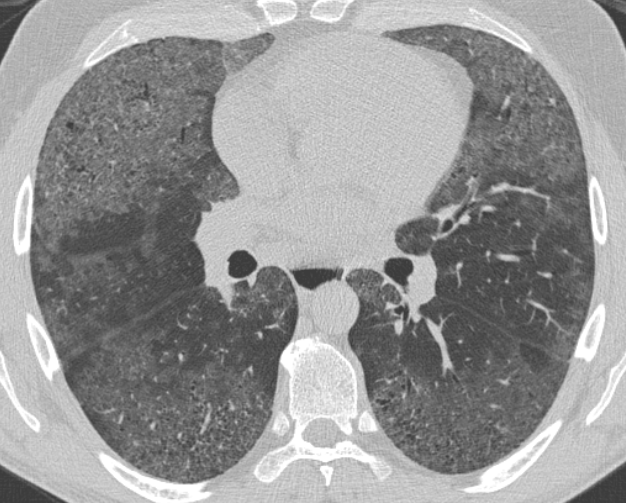

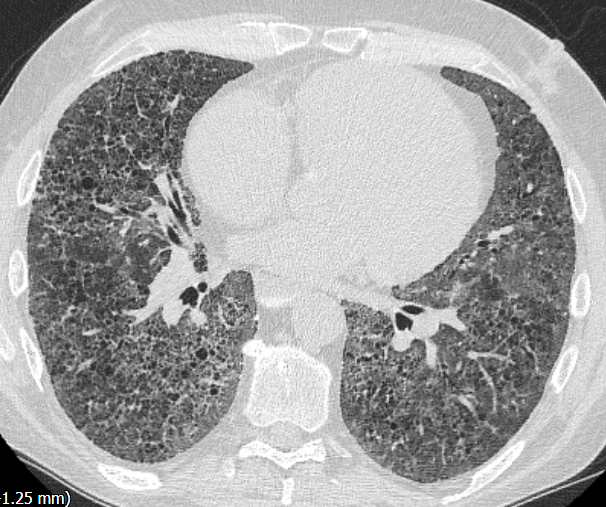


**A**

**Baseline**

**179 months**


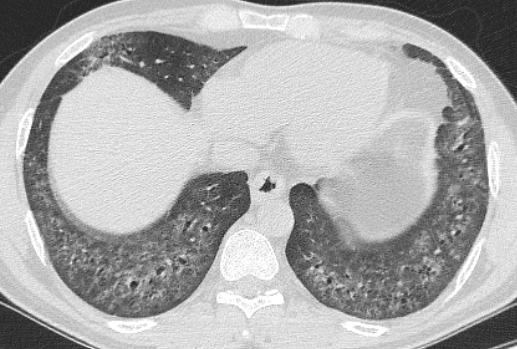

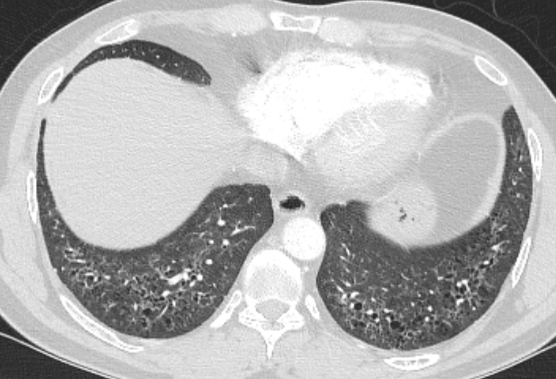


**B**

**Baseline**

**33 months**


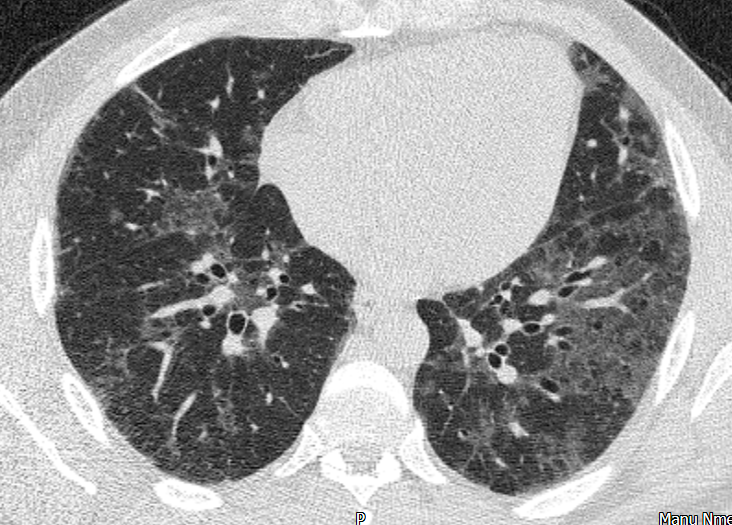

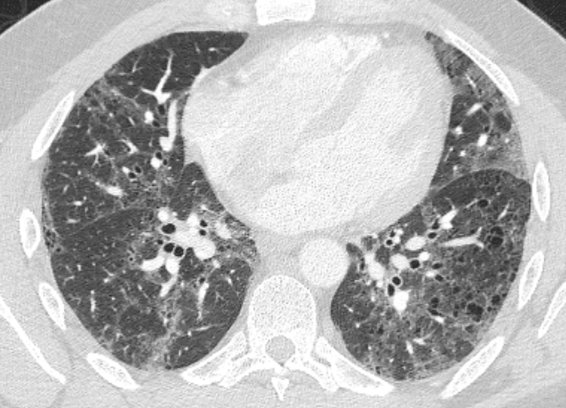


**C**

**Baseline**

**29 months**


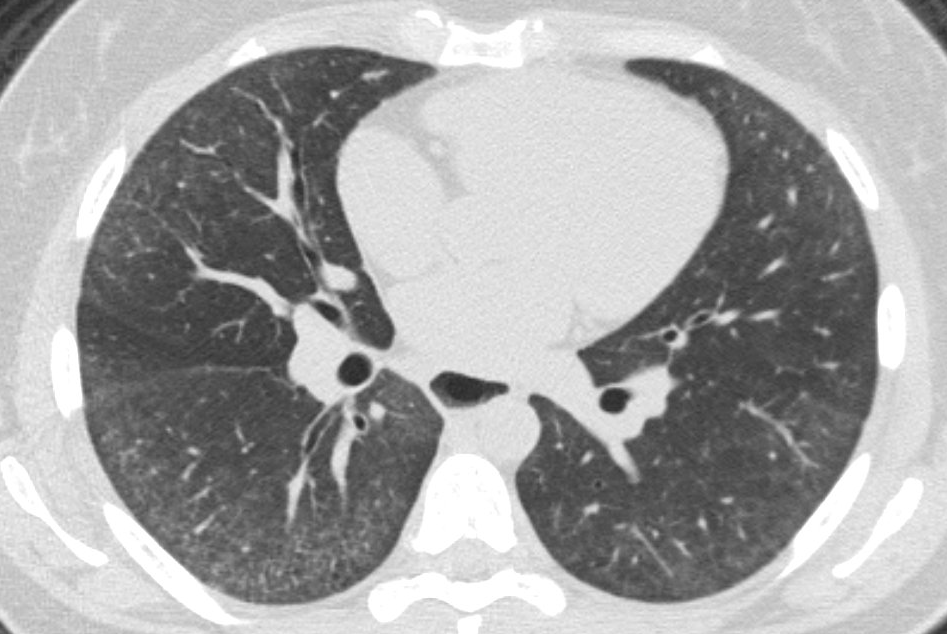

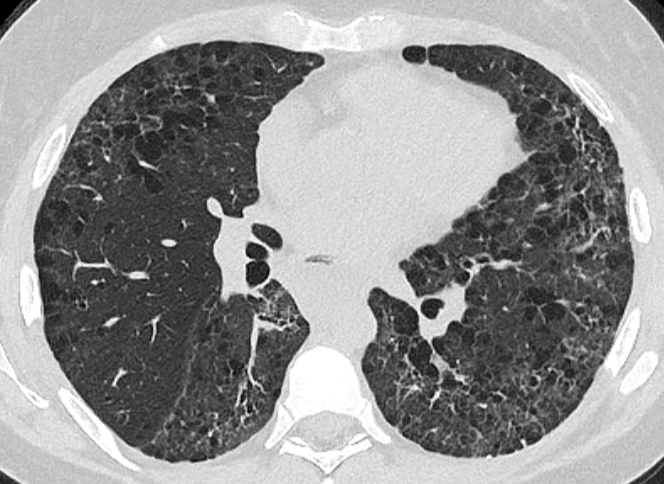


**D**

**Baseline**

**58 months**


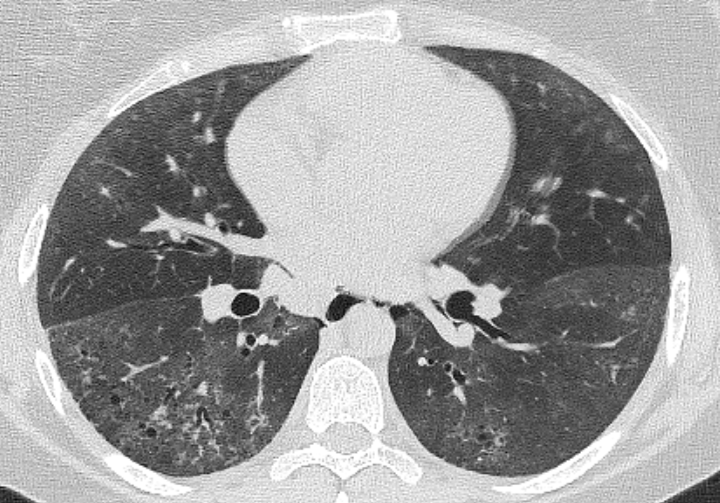

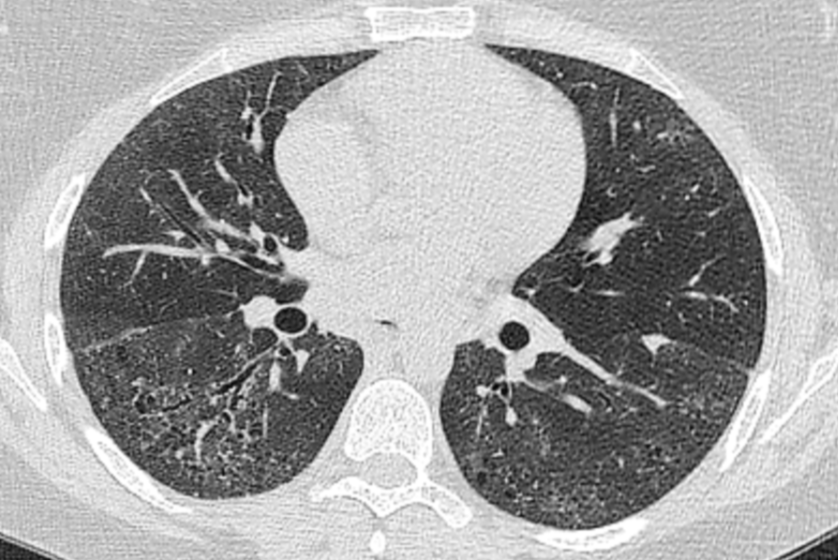


**E**

**Baseline**

**10 months**


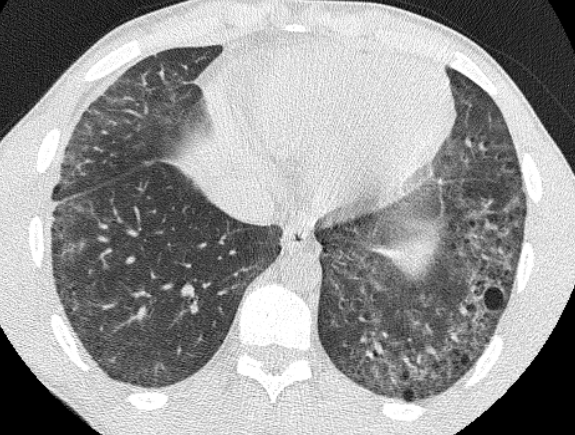

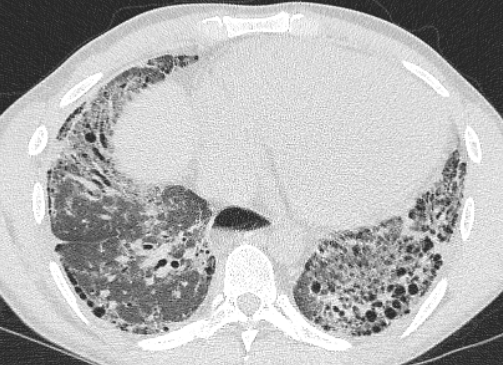


**F**

**Baseline**

**126 months**


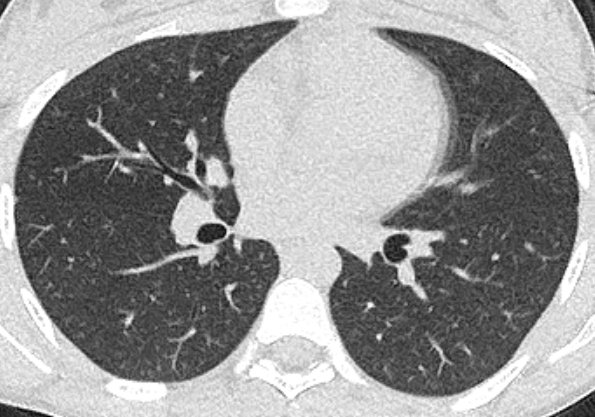

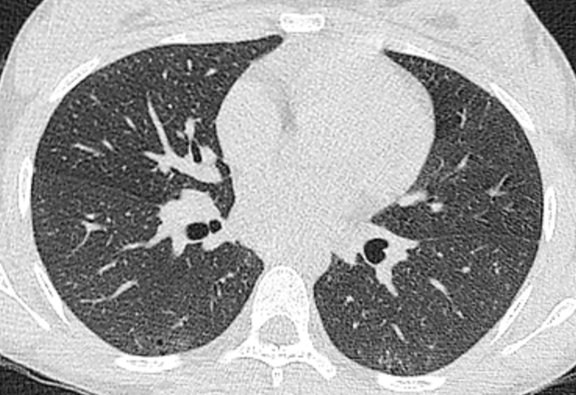


**G**

**Baseline**

**34 months**

Supplementary Figure S3. Unannotated HRCT images. See figure 1 (main document) for legend.
